# Arteriolar vasoconstrictive response: comparing the effects of arginine vasopressin and norepinephrine

**DOI:** 10.1186/cc4922

**Published:** 2006-05-12

**Authors:** Barbara E Friesenecker, Amy G Tsai, Judith Martini, Hanno Ulmer, Volker Wenzel, Walter R Hasibeder, Marcos Intaglietta, Martin W Dünser

**Affiliations:** 1 Division of General and Surgical Intensive Care Medicine, Department of Anesthesiology and Critical Care Medicine, Innsbruck Medical University, Innsbruck, Austria; 2 Department of Bioengineering, University of California, San Diego, CA, USA; 3 Institute of Biostatistics and Documentation, Medical University Innsbruck, Innsbruck, Austria; 4 Division of Anesthesiology, Department of Anesthesiology and Critical Care Medicine, Innsbruck Medical University, Innsbruck, Austria; 5 Department of Anesthesiology and Critical Care Medicine, Krankenhaus der Barmherzigen Schwestern, Ried im Innkreis, Austria; 6 Department of Intensive Care Medicine, University Hospital of Bern, Bern, Switzerland

## Abstract

**Introduction:**

This study was designed to examine differences in the arteriolar vasoconstrictive response between arginine vasopressin (AVP) and norepinephrine (NE) on the microcirculatory level in the hamster window chamber model in unanesthetized, normotonic hamsters using intravital microscopy. It is known from patients with advanced vasodilatory shock that AVP exerts strong additional vasoconstriction when incremental dosage increases of NE have no further effect on mean arterial blood pressure (MAP).

**Methods:**

In a prospective controlled experimental study, eleven awake, male golden Syrian hamsters were instrumented with a viewing window inserted into the dorsal skinfold. NE (2 μg/kg/minute) and AVP (0.0001 IU/kg/minute, equivalent to 4 IU/h in a 70 kg patient) were continuously infused to achieve a similar increase in MAP. According to their position within the arteriolar network, arterioles were grouped into five types: A0 (branch off small artery) to A4 (branch off A3 arteriole).

**Results:**

Reduction of arteriolar diameter (NE, -31 ± 12% versus AVP, -49 ± 7%; *p *= 0.002), cross sectional area (NE, -49 ± 17% versus AVP, -73 ± 7%; *p *= 0.002), and arteriolar blood flow (NE, -62 ± 13% versus AVP, -80 ± 6%; *p *= 0.004) in A0 arterioles was significantly more pronounced in AVP animals. There was no difference in red blood cell velocities in A0 arterioles between groups. The reduction of diameter, cross sectional area, red blood cell velocity, and arteriolar blood flow in A1 to A4 arterioles was comparable in AVP and NE animals.

**Conclusion:**

Within the microvascular network, AVP exerted significantly stronger vasoconstriction on large A0 arterioles than NE under physiological conditions. This observation may partly explain why AVP is such a potent vasopressor hormone and can increase systemic vascular resistance even in advanced vasodilatory shock unresponsive to increases in standard catecholamine therapy.

## Introduction

Since its first detection in 1895 by Schaefer and Oliver [1], arginine vasopressin (AVP) has been known for its potent vasoconstrictive effects. During the past decade, successful clinical application of AVP has been reported in cardiac arrest [2] and advanced vasodilatory shock [3]. In all of these diseases, AVP can exert strong vasoconstriction and significantly increase perfusion pressure even in shock states when standard catecholamine therapy could not control vascular tone. These clinical observations unequivocally support the physiological finding that, on a molar basis, AVP is a several fold stronger vasopressor hormone than angiotensin II, epinephrine, or norepinephrine (NE) [4], although its mechanisms of action are unclear.

Stimulation of V_1a_-receptors located on vascular smooth muscle of arterioles mediates contraction and thereby causes vasoconstriction [5]. Nonetheless, although repeatedly proven in the clinical setting, it remains unknown why AVP can still cause a significant increase in vascular tone when stimulation of α-adrenergic receptors fails to increase perfusion pressure. Several hypotheses have suggested that additional pharmacological effects of AVP, such as inhibition of activated K_ATP_-channels or endothelial nitric oxide synthase, and synergistic effects between catecholamines and AVP may explain AVP's potent vasoconstrictive effects [6]. However, the mechanism of nitric oxide inhibition by AVP, for example, has recently been proven to play only a minor or irrelevant role in the clinical setting [7]. This experimental study was designed to evaluate differences in the arteriolar vasoconstrictive response between AVP and NE in a physiological hamster model [8]. Our hypothesis was that there were no differences in the arteriolar vasoconstrictive response between AVP and NE.

## Materials and methods

### Animal model and preparation

The experimental protocol was approved by the Austrian Ministry of Science and Research. While the animals were under intraperitoneal pentobarbital anesthesia (50 mg/kg body weight), a viewing window was inserted into the dorsal skinfold of 11 male golden Syrian hamsters (weight 60 to 85 g; Charles River Laboratories, Sulzfeld, Germany) [9]. Briefly, the dorsal skinfold consisting of two layers of skin and corresponding muscle tissue was placed between two titanium frames. A 15 mm circular portion of the skin, including two skin muscles with the underlying skin, remained in place. The tissue was covered with saline, and a cover glass was held by one side of the titanium frame, yielding a stable preparation that allows repeated microscopic observations over several days. The area of microscopic observation is originally located just behind the large front vessels that feed and drain the chamber network. A modified preparation technique was used where the tissue studied is nearer to the animal's head to allow microscopic observation of the large feeding arteriole (A0) of the chamber network [10]. Two days after chamber implantation, polyethylene-50 catheters were inserted into the internal carotid artery and external jugular vein for evaluation of systemic parameters (mean arterial blood pressure (MAP), heart rate) and infusion of study drugs.

### Inclusion criteria

**Figure 1:**
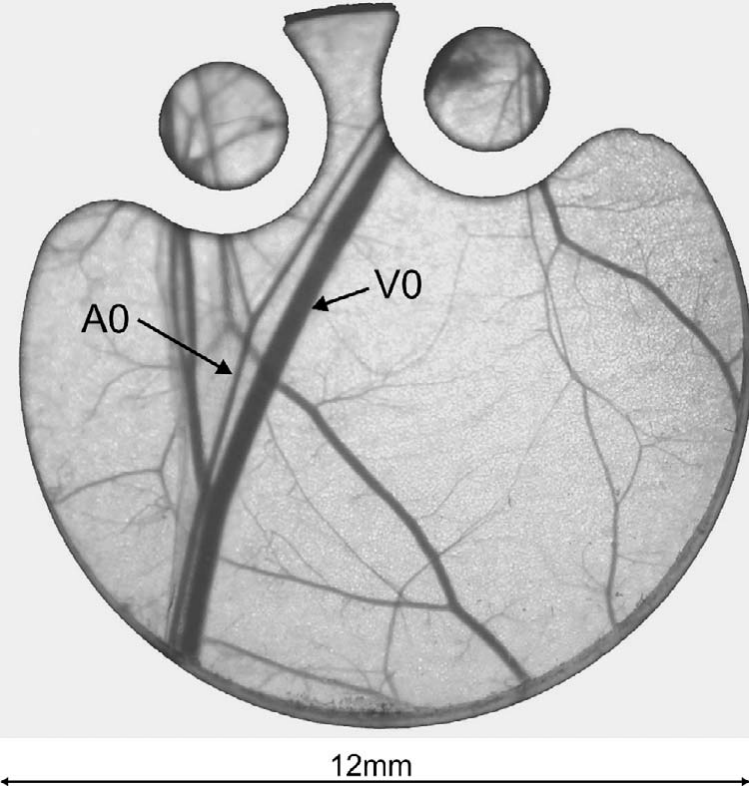
Hamster window chamber model. *In-vivo *preparation of the hamster window chamber model with visible A0 arteriole and V0 vein. Other vessels (A1, branch off A0; A2, branch off A1; A3, branch off A2; A4, branch off A3), capillaries (defined as vessels with single red cell transit), and venules can only be classified under the intravital microscope.

Animals were eligible for inclusion into the study protocol if their systemic parameters were within normal range, namely heart rate >340 beats per minute and MAP >80 mmHg, and microscopic examination of the tissue in the chamber observed under ×600 magnification did not reveal signs of edema or bleeding (Figure 1).

### Systemic parameters

MAP was tracked periodically during the experiment through the arterial catheter, and heart rate was determined from the pressure trace (Recom pressure transducer system, model 13-6615-50, Gould Instrument Systems, Ohio, USA).

### Arteriolar vasoconstrictive response

Arteriolar diameters (D) were measured using the video image shearing technique (model 908, Vista Electronics, San Diego, CA, USA). Cross-sectional areas of arterioles were calculated according to standard mathematical formulas. The measured centreline velocity (V) was corrected according to vessel size to obtain the mean velocity of red blood cells. Arteriolar blood flow (Q) was calculated according to the formula [11]:

Q = V × π × (D^2 ^× 0.001^2^/4)

Depending on their position within the microvascular network, arterioles were grouped into five categories: A0 arteriole, branch off small artery; A1-arteriole, branch off A0; A2 arteriole, branch off A1; A3 arteriole, branch off A2; A4 arteriole, branch off A3 (Figure 1).

### Experimental setup

An unanesthetized animal was placed in a restraining tube that was stabilized by affixing the tube and the chamber to a Plexiglas plate. The animal had free access to wet feed during the entire experimental period. The Plexiglas stage that held the animal was then placed on an intravital microscope (Mikron Instruments, San Diego, CA, USA) equipped with a F0-150 halogen fiberoptic illuminator (CHIU Technical, Kings Park, NY, USA) and two infinity-corrected objectives (Zeiss Achroplan ×20/0.5 W, ×40/0.75 W). A 420 nm blue filter was used for contrast enhancement of the transilluminated image. The image was projected onto a charge-coupled device camera (model COHU FK 6990 IQ-S, Pieper; Düsseldorf, Germany) and viewed on a monitor (model PVM-1454QM, Sony). The animal was allowed a 30 minute adjustment period to the tube environment before baseline measurements. Microvascular fields of study were chosen by their visual clarity.

### Study protocol and drug dosage

Study animals were randomly assigned to a NE and an AVP group. Animals in the AVP group received a continuous infusion of AVP at a clinically relevant dosage of 0.0001 IU/kg/minute (corresponding to 4 IU/h in a 70 kg critically ill patient [3,12]) throughout the time of the experiment.

In a small pilot study, this dosage was found to attain a consistent and stable level of vasoconstriction. In contrast, half of this AVP dosage (0.00005 IU/kg/minute) did not cause a relevant change in mean arterial pressure. Infusion of ten times the higher AVP dosage (0.001 IU/kg/minute) resulted in a comparable increase in mean arterial pressure, but caused a microcirculatory 'low flow state', and even stopped arteriolar blood flow in one pilot animal. According to the chosen AVP dosage of 0.0001 IU/kg/minute, the NE dosage of 2 μg/kg/minute was determined to achieve a similar increase in MAP.

In all animals, the infusion volume was calculated not to exceed 10% of blood volume in each individual animal. After taking control measurements, the study drug was infused over 30 minutes before systemic and microvascular measurements were performed during continuous study drug infusion.

### Statistical analysis

The study endpoint was to evaluate differences in the arteriolar vasoconstrictive response between NE- and AVP-treated animals.

Shapiro Wilk's and Kolmogorov Smirnov tests were used to check for normal distribution of data. Because normality assumption was not fulfilled in main study variables, non-parametric tests (Mann Whitney *U* rank sum test) were applied for comparisons between study groups at baseline and within repeated measurements. The same tests were used to detect significant changes during drug infusion when compared to baseline within groups. For comparison within the five arteriolar subgroups, Bonferroni corrections for multiple comparisons were applied, and the significance level was set at 0.01. Study results are given as mean values ± standard deviations, if not indicated otherwise.

## Results

Eleven animals met the study inclusion criteria and were entered into the randomization process (NE, *n *= 5; AVP, *n *= 6). All animals completed the study protocol without visible signs of discomfort. Animals were observed resting and periodically eating throughout the experiment.

No statistically significant differences were observed in systemic or microvascular variables measured at study entry between groups.

### Systemic parameters

In pilot studies NE dosage was chosen to match AVP induced MAP changes. During the experiment, infusion of NE and AVP caused both a significant increase in MAP and a significant decrease in heart rate (Table 1). These changes were not different between study groups (heart rate, *p *= 0.221; MAP, *p *= 0.847).

### Microvascular parameters

**Figure 2:**
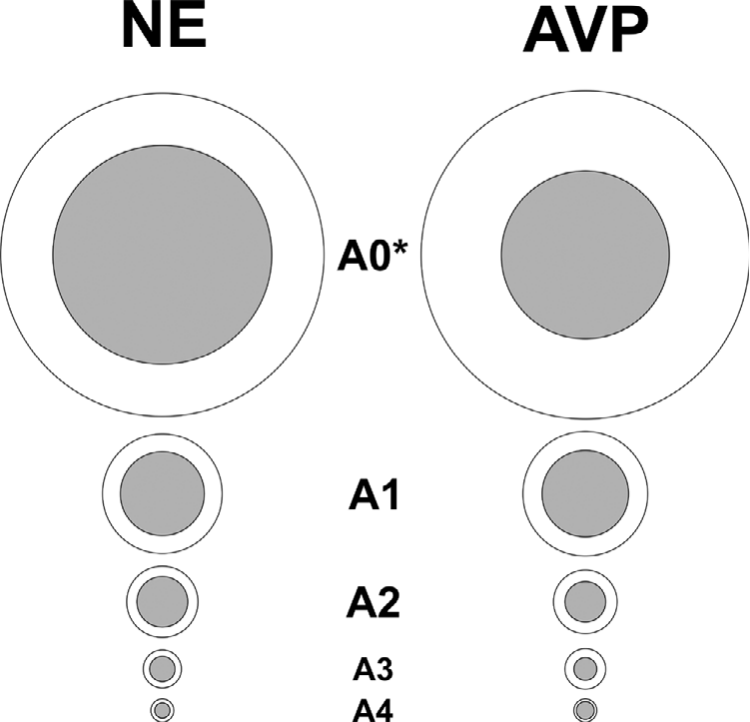
Cross-sectional arteriolar areas. Differences in cross-sectional area (μm^2^) of A0, A1, A2, A3 and A4 arterioles between norepinephrine (NE) and arginine vasopressin (AVP) treated animals (drawn true to scale). The asterisk indicates a significant difference between groups (*p *< 0.002).

In A0 arterioles, the reduction of diameter and cross sectional area was more pronounced in AVP animals when compared to NE-treated animals (Table 2 and Figure 2). Accordingly, arteriolar flow was significantly more reduced in AVP animals than in the NE group. There were no differences in red blood cell velocity in A0 arterioles between study groups.

In A1 to A4 arterioles, there were no differences in arteriolar diameter or cross-sectional area between AVP and NE animals. Neither red blood cell velocity nor arteriolar blood flow were significantly different between the two study groups.

## Discussion

In this animal experiment, the reduction of arteriolar diameter, cross-sectional area, and arteriolar blood flow was significantly different between NE and AVP animals under physiological conditions. AVP-treated animals exhibited a significantly greater vasoconstrictive response in large A0 arterioles when compared to NE animals, while there was no difference in A1 to A4 arterioles between study groups.

The greater decrease in arteriolar diameter and cross-sectional area of A0 arterioles during AVP infusion when compared to NE therapy clearly indicates that AVP exerted significantly stronger vasoconstrictive effects on large arterioles, which ultimately control blood flow to the subsequent vessels of the microcirculatory system. Although receptors have not been assessed quantitatively or qualitatively in this experiment, it may be hypothesized that relatively more V_1a_- than α-receptors are located on vascular smooth muscle of A0 arterioles. Nonetheless, it cannot be excluded that specific receptor-independent AVP effects on vascular tone, such as inhibition of K_ATP_-potassium channels [13], contributed to strong vasoconstriction induced by AVP in A0 arterioles as well.

This is the first study identifying a significant difference in the arteriolar vasoconstrictive response between AVP and an adrenergic vasopressor agent on the microcirculatory level under primarily physiological conditions. To the best of our knowledge, it is also the first experiment to observe that AVP, in comparison to NE, exerts significantly stronger vasoconstriction in large arterioles. So far, only one study has examined the arteriolar vasoconstriction pattern after injection of AVP. Marshall and colleagues [14] reported strong AVP-mediated vasoconstrictive effects on proximal arterioles of the spinotrapezius muscle of the rat. Important differences to our study protocol were that arterioles were grouped only in a proximal (>13 μm) and a distal (<13 μm) group, and there was no comparison with an adrenergic vasopressor agent. Additionally, study animals received AVP as a bolus injection, and were hypoxic and anesthetized; all factors that may have influenced or altered AVP-mediated vasoconstriction. Interestingly, the same authors observed that vasoconstriction exerted by NE during hypoxia was most pronounced in arteriolar vessels measuring 13 to 50 μm in diameter [15], corresponding to the more recent definition of A2 to A4 arterioles, which is in accordance with the results of our experiment. In an anesthetized rat model, Baker and colleagues [16] similarly observed that large arterioles (approximately 130 to 110 μm) exhibited significantly stronger constriction when compared to smaller arterioles (approximately 40 μm) in the cremaster muscle after topical application of AVP.

It is well known that changes in arteriolar tone mainly contribute to the regulation of systemic vascular resistance and thus arterial blood pressure [17]. While earlier studies have focused on the behavior of A2 to A4 arterioles, it has been shown in hypertensive rats that large arterioles and small arteries, and not small arterioles, are primarily responsible for changes in systemic vascular resistance [18,19]. In a dorsal skin flap preparation in rats, le Noble and colleagues [20] concluded that, in the established phase of spontaneous hypertension, a decreased diameter of large arterioles was the major mechanism underlying the increase in vascular resistance. Similarly, Grega and colleagues [21] suggested that small arteries and larger arterioles may contribute more than smaller arterioles to increases in systemic vascular resistance produced by local infusion of vasopressor agents. Additionally, in conscious hamsters with hemorrhagic shock, vasoconstriction was found to be strongest in A0 arterioles, while smaller arterioles exhibited only small diameter changes or, under some conditions, even vasodilation [10].

These observations in physiological and pathophysiological models match the findings of the present study where AVP constricts larger arterioles to a significantly greater extent than NE and may explain why AVP is able to induce a more significant increase in systemic vascular resistance than other adrenergic vasopressor hormones [4]. Moreover, these results may partly elucidate the finding that AVP given as a continuous infusion can increase arterial pressure even in advanced vasodilatory shock states unresponsive to standard hemodynamic therapy, including infusion of NE [3,12,22].

Corresponding to the pronounced reduction of arteriolar diameter and cross-sectional area, blood flow was significantly more reduced in A0 arterioles in AVP-treated animals then in the NE-group. Interestingly, however, blood flow was not decreased in successive A1 to A4 arterioles during AVP infusion when compared to NE infusion. This is particularly striking, since one would expect a similarly pronounced reduction of arteriolar blood flow in all consecutive arterioles in the face of significantly reduced inflow in the main feeding arteriole. While A0 arterioles obviously contribute significantly to systemic vascular resistance, their influence on arteriolar blood flow seems to be less pronounced, at least in our experiment. This finding again corresponds to the clinical observation that despite a significant increase in systemic vascular resistance in patients with advanced vasodilatory shock receiving a supplementary AVP infusion, end-organ perfusion is not impaired when compared to patients with high dose NE therapy alone [3,12,22].

When interpreting the results of this study, and particularly when drawing conclusions for the clinical setting, important limitations need to be noted. First, since the present study was designed to examine differences in the arteriolar vasoconstrictive response between AVP and NE under physiological conditions, further research needs to be conducted to elucidate whether the observed microcirculatory response to AVP and NE follows a comparable pattern under pathophysiological conditions such as vasodilatory shock. Second, in contrast to our study in animals, most critically ill patients with advanced vasodilatory shock are ventilated and sedated. From animal experiments, it is well known that infusion of sedative drugs, for example, pentobarbital, causes a significant reduction of microvascular blood flow of the arteriolar and venular system as well as a decrease in functional capillary density [23]. Third, as the vasoconstrictive response to AVP has been reported to differ between some vascular beds and certain species [24,25], the results of this study cannot be simply transferred into the clinical setting. However, since arterioles in the skin and musculature significantly contribute to changes in systemic vascular resistance [17], the skin might very well be a key organ to primarily assess and compare the vasoconstrictive potency of vasopressor agents.

## Conclusion

Under physiological conditions, AVP exerted significantly stronger vasoconstrictive effects on large arterioles than NE in this hamster window chamber model. This observation may partly explain why AVP is such a potent vasopressor hormone and can increase systemic vascular resistance beyond the level of standard catecholamine therapy in advanced vasodilatory shock states.

## Key messages

• 	The higher vasoconstrictive potency of AVP when compared to NE may be partly explained by a significantly more pronounced vasoconstriction of large arterioles within the microvascular bed of the hamster skinfold under physiological conditions.

## Abbreviations

AVP = arginine vasopressin; MAP = mean arterial blood pressure; NE = norepinephrine.

## Competing interests

The authors declare that they have no competing interests.

## Authors' contributions

BF, AT, JM and MD designed the study protocol and drafted the manuscript. BF, AT, JM performed the animal surgery and carried out the experiments. HU helped with the study design and statistical evaluation. VW, WH, MI, MD made substantial contributions to conception and design as well as analysis of data and have been involved in revising the mansucript for intellectual content. All authors gave final approval of the version to be published.

## Figures and Tables

**Table 1:** Heart rate and mean arterial pressure in norepinephrine and arginine vasopressin treated animals

	Baseline	Drug infusion	*p *value^a^
Heart rate (bpm)			
NE^b^	449 ± 25	399 ± 44	0.847
AVP^b^	452 ± 36	403 ± 44	
MAP (mmHg)			
NE^b^	103 ± 8	129 ± 7	0.221
AVP^b^	98 ± 10	121 ± 8	

Data are given as mean values ± standard deviation. ^a^*P *value for differences between groups. ^b^Significant difference between baseline and drug infusion. AVP, arginine vasopressin; bpm, beats per minute; MAP, mean arterial blood pressure; NE, norepinephrine.

**Table 2:** Arteriolar diameter, cross-sectional area, blood velocity, and arteriolar flow in norepinephrine and arginine vasopressin animals

Arteriol type	Parameter	Drug	Baseline	Drug infusion	Change (%)	*p *value
A0	Arteriolar D (μm)	NE^a^	127 ± 27	86 ± 16	31 ± 12	0.002^b^
		AVP^a^	129 ± 7	66 ± 12	49 ± 7	
	Arteriolar CSA (μm^2^)	NE^a^	13,083 ± 4,908	5,954 ± 2,150	49 ± 17	0.002^b^
		AVP^a^	13,100 ± 1,462	3,547 ± 1,173	73 ± 7	
	RBC velocity (mm/s)	NE^a^	13.5 ± 2.2	10.6 ± 2.3	22 ± 9	0.232
		AVP^a^	14.9 ± 0.9	11.2 ± 1.1	25 ± 6	
	Arteriolar BF (10^-2 ^× mm × μm^2^/s)	NE^a^	18.4 ± 8.1	6.6 ± 3.2	62 ± 13	0.004^b^
		AVP^a^	19.6 ± 2.6	3.9 ± 1.3	80 ± 6	
A1	Arteriolar D (μm)	NE^a^	47 ± 11	33 ± 8	28 ± 12	0.461
		AVP^a^	49 ± 13	66 ± 12	30 ± 12	
	Arteriolar CSA (μm^2^)	NE^a^	1,785 ± 878	922 ± 444	47 ± 16	0.461
		AVP^a^	2,000 ± 1,080	987 ± 574	50 ± 17	
	RBC velocity (mm/s)	NE^a^	3.7 ± 0.7	3 ± 0.4	19 ± 9	0.236
		AVP^a^	3.9 ± 1	2.9 ± 1.2	27 ± 17	
	Arteriolar BF (10^-3 ^× mm × μm^2^/s)	NE^a^	6.9 ± 4.1	2.8 ± 1.5	57 ± 15	0.096
		AVP^a^	8.2 ± 6.2	2.9 ± 2.4	63 ± 14	
A2	Arteriolar D (μm)	NE^a^	28 ± 12	20 ± 9	29 ± 12	0.156
		AVP^a^	25 ± 8	16 ± 5	34 ± 14	
	Arteriolar CSA (μm^2^)	NE^a^	748 ± 689	390 ± 332	48 ± 16	0.156
		AVP^a^	522 ± 345	226 ± 141	55 ± 18	
	RBC velocity (mm/s)	NE^a^	3 ± 0.7	2.3 ± 0.4	20 ± 14	0.845
		AVP^a^	2.8 ± 0.4	2.2 ± 0.4	21 ± 15	
	Arteriolar BF (10^-3 ^× mm × μm^2^/s)	NE^a^	2.6 ± 3.3	0.9 ± 1.0	59 ± 13	0.212
		AVP^a^	1.5 ± 1.2	0.5 ± 0.4	64 ± 19	
A3	Arteriolar D (μm)	NE^a^	15 ± 6	10 ± 5	34 ± 11	0.110
		AVP^a^	16 ± 5	9 ± 3	43 ± 12	
	Arteriolar CSA (μm^2^)	NE^a^	193 ± 180	86 ± 96	56 ± 14	0.110
		AVP^a^	86 ± 96	74 ± 51	86 ± 13	
	RBC velocity (mm/s)	NE^a^	2.3 ± 0.4	1.8 ± 0.4	21 ± 16	0.146
		AVP^a^	2.4 ± 0.4	1.7 ± 0.6	29 ± 16	
	Arteriolar BF (10^-4 ^× mm × μm^2^/s)	NE^a^	4.7 ± 4.9	1.6 ± 1.6	65 ± 12	0.013
		AVP^a^	5.6 ± 4.9	1.3 ± 1.3	76 ± 11	
A4	Arteriolar D (μm)	NE^a^	9 ± 3	6 ± 1	32 ± 8	0.206
		AVP^a^	9 ± 2	7 ± 2	26 ± 12	
	Arteriolar CSA (μm^2^)	NE^a^	70 ± 62	28 ± 14	53 ± 10	0.206
		AVP^a^	74 ± 41	39 ± 20	44 ± 18	
	RBC velocity (mm/s)	NE^a^	1.7 ± 0.3	1.3 ± 0.3	21 ± 14	0.464
		AVP^a^	1.5 ± 0.3	1.1 ± 0.1	27 ± 19	
	Arteriolar BF (10^-4 ^× mm × μm^2^/s)	NE^a^	1.3 ± 1.4	0.4 ± 0.3	63 ± 10	0.837
		AVP^a^	1.2 ± 0.9	0.5 ± 0.3	57 ± 21	

Data are given as mean values ± standard deviation. ^a^Significant difference between baseline and drug infusion. ^b^Significant difference of change (%) between arginine vasopressin (AVP) and norepinephrine (NE) animals. BF, blood flow; CSA, cross-sectional area; D, diameter; RBC, red blood cell.
